# Potential vertical transmission of genetically diverse *Trypanosoma cruzi* in natural rodent populations

**DOI:** 10.1371/journal.pntd.0012930

**Published:** 2025-04-01

**Authors:** Nathaniel L. Gibson, Bruno M. Ghersi, Bridget Knudson, Anna C. Peterson, Claudia Riegel, Weihong Tu, Eric Dumonteil, Claudia Herrera, Michael J. Blum

**Affiliations:** 1 University of Tennessee Knoxville, Knoxville, Tennessee, United States of America; 2 Tufts University Cummings School of Veterinary Medicine, North Grafton, Massachusetts, United States of America; 3 Tulane University, Celia Scott Weatherhead School of Public Health and Tropical Medicine, New Orleans, Louisiana, United States of America; 4 City of New Orleans Mosquito, Termite, & Rodent Control Board, New Orleans, Louisiana, United States of America; U.S. Food and Drug Administration and Center for Biologics Evaluation and Research, UNITED STATES OF AMERICA

## Abstract

**Background:**

*Trypanosoma cruzi*, the causative agent of Chagas disease, has been detected in mammalian hosts occupying densely populated urban environments. This suggests that the risk of transmission to humans is higher than prevailing estimates, which largely reflect conditions in rural and peri-urban areas. Understanding the risks posed by *T. cruzi* thus requires further study of transmission pathways in part because triatomines – the primary vectors for *T. cruzi* – appear to be uncommon or absent in urban landscapes. Here we test the hypothesis that vertical transmission contributes to the prevalence of infection and diversity of *T. cruzi* in urban reservoirs.

**Methodology and Principal Findings:**

We assessed whether embryos of *T. cruzi*-positive parous female rodents also exhibit evidence of infection. A diagnostic PCR assay detected *T. cruzi* in 15 out of 66 (22.7%) embryos from Norway rats, black rats, and house mice captured in New Orleans (LA, USA). Genotyping PCR identified the presence of TcI and non-TcI discrete typing units (DTUs) in individual infected embryos, providing evidence of mixed infection. Next-generation sequencing provided additional evidence of mixed infection in individual embryos.

**Conclusions:**

Our findings provide additional evidence that vertical transmission can occur in natural populations of reservoir species and demonstrates for the first time that multiple DTUs can transmit from mother to offspring. Our study also demonstrates that vertical transmission can contribute to the prevalence of infection and diversity of *T. cruzi* in multiple reservoir species occupying urban landscapes where vectors appear to be rare or absent, providing a new baseline for understanding transmission pathways and eco-epidemiological cycling of *T.cruzi.*

## Author summary

*Trypanosoma cruzi*, the parasite that causes Chagas disease, has recently been detected in commensal hosts across cities where kissing bug vectors can be rare or absent. This suggests that a different mode of transmission is sustaining the parasite in cities. Here we show that *T. cruzi* is being passed from mother to offspring in natural populations of Norway rats, black rats, and house mice in New Orleans, Louisiana (USA). PCR-based testing of embryos from infected mothers showed that about a quarter of offspring were infected with *T. cruzi*. Molecular diagnostics also provided evidence of mixed infection in individual embryos, indicating that vertical transmission can contribute to *T. cruzi* infection prevalence and diversity in urban reservoirs. These findings set new baselines for determining risks posed by *T. cruzi* to humans in cities and other areas where it is widespread.

## Introduction

The protozoan parasite *Trypanosoma cruzi* is the etiological agent of Chagas disease, often described as the most burdensome anthropozoonosis in the Western Hemisphere [1,2]. An estimated 6–7 million people have developed Chagas disease due to *T. cruzi* infection and upwards of 100 million people are at risk of infection. Chronic infection can result in damage to digestive systems (e.g., enlarged esophagus and/or colon) and cardiovascular damage (e.g., cardiomyopathy), which can lead to death. While the majority of human infections occur in Mexico, Central and South America, *T. cruzi* vectors and hosts are endemic to the southern United States [1,3]. Aspects of global change like increasing temperature and land use intensification as well as immigration and displacement of human populations are expected to expand the range of *T.* cruzi in North America [4–7]. It also appears that *T. cruzi* is expanding into novel landscapes. Notably, the long-standing convention that Chagas disease is a rural disease has been challenged by recent studies showing that *T. cruzi-*infected hosts can be widespread across urban landscapes [8–11]. Urban hosts also appear to harbor genetically diverse assemblages of *T. cruzi* DTUs [12], further suggesting that risk of transmission to humans is far higher than currently thought.

Evidence of infection in low-vagility hosts like Norway rats (*Rattus norvegicus*) and other commensal rodents in cities (e.g., [8]) suggests that *T. cruzi* is primarily sustained in urban reservoirs by local transmission via resident triatomine vectors. Some mammalian hosts (e.g., raccoons, feral dogs) that frequent urban landscapes are highly mobile, however, and thus could be infected upon contact with triatomines in proximate peri-urban or sylvatic habitats [13,14]. Such incidental transmission is unlikely to occur with low-vagility hosts like Norway rats, which rarely move more than a distance equivalent to a city block over the course of a lifespan [15,16]. This would suggest that contact with triatomine vectors is local and widespread. Yet work thus far indicates that triatomines are uncommon- and perhaps absent- across urban landscapes [17,18]. It follows then that other transmission pathways could be maintaining *T. cruzi* in urban reservoirs.

Vertical transmission, wherein infection is passed from mother to offspring, represents a potential pathway for maintaining *T. cruzi* in reservoirs where vectors are rare or absent. Instances of vertical transmission have been widely documented in cases of human infection [19,20], and laboratory-based studies have demonstrated that vertical transmission can occur in rodent hosts [21–23]. So far, however, laboratory-based studies of vertical transmission have not considered several key aspects of infection that can contribute to the maintenance of *T. cruzi* in natural reservoirs. For example, laboratory tests have focused on transmission of a single discrete typing unit (DTU) of *T. cruzi*. Recent work suggests that diverse assemblages of DTUs can co-occur in a host (hereafter referred to as ‘mixed infection’) [24], and that mixed infection is likely common in rodents [12,25]. It is also unclear whether vertical transmission varies among hosts, with conveyance from mother-to-offspring occurring more frequently in some species more than others. Vertical transmission thus merits further study. A focus on natural populations of low-vagility urban hosts would be particularly informative, as finding evidence of vertical transmission would indicate that it might sustain infection and genetic variation of *T. cruzi* in reservoirs that have little or no contact with triatomine vectors.

We leveraged an existing archive of *T. cruzi*-positive rodents from metropolitan New Orleans (LA, USA) [8,26] to determine the extent of vertical transmission within and among species of an urban rodent community. By drawing comparisons between infected mothers and their embryos, respectively, we first determined whether there is evidence of vertical transmission (i.e., presence of *T. cruzi* in embryos from *T. cruzi*-positive mothers) and if so, how transmission frequency compares within and among host species. We also compared DTU profiles of infected mothers and embryos to determine whether and how vertical transmission might influence *T. cruzi* diversity in rodent hosts and host populations. If conveyance varies among DTUs, for example, then vertical transmission might act as a filter that reduces the prevalence of mixed infection and *T. cruzi* diversity within host populations. We expected to find rates of vertical transmission comparable to those observed in laboratory settings, though we anticipated that mixed infection is less likely in embryos due in part to the possibility of tissue tropism limiting *T. cruzi* infection (i.e., a lack of tissue specificity in embryos leads to less opportunity for mixed infection) [12,24]. Accordingly, we anticipated that infected embryos would carry the most prevalent DTU (i.e., TcI) so far detected in rodents from the study area [25].

## Methods

### Ethics statement

Sampling protocols were approved by the Tulane University Instituational Animal Care & Use Committee (IACUC) protocols #0451 and #0460.

### Study area and samples

**Fig 1:**
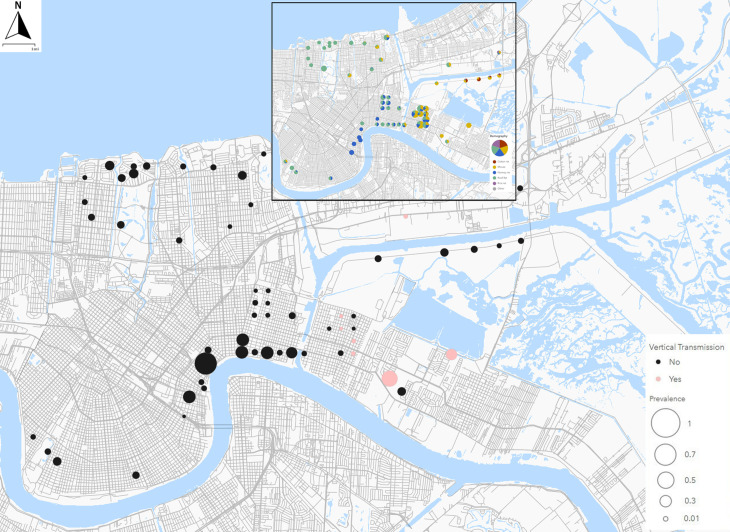
Map of T. cruzi prevalence in rodents across New Orleans and neighboring St. Bernard Parish. Black dots identify sites where T. cruzi-positive rodents were located with dot size representing total prevalence of infection; red dots identify sites with T. cruzi-positive parous mothers and embryo(s). The inset map describes the rodent assemblage at sites with T. cruzi infected rodents with colors denoting rodent species, and size representing number of rodents collected. Maps were created using ArcGIS Online (https://www.arcgis.com/index.html), utilizing layers from Natural Earth (https://www.naturalearthdata.com/downloads/10m-physical-vectors/) and US Census Bureau Tiger/LINE shapefiles (road - https://www.census.gov/cgi-bin/geo/shapefiles/index.php?year=2024&layergroup=Roads; water - https://www.census.gov/cgi-bin/geo/shapefiles/index.php?year=2024&layergroup=Water).

All parous females used in this study were trapped as part of quantitative demographic and assemblage surveys in Orleans Parish and an adjacent area of St. Bernard Parish (LA, USA) between May 2014 and February 2017 as detailed in Peterson et al. [27] and Ghersi et al. [28] (Fig 1). All specimens and associated tissue samples have been stored at -80^o^ C since the time of original necropsy. PCR assays of genomic DNA from blood or heart tissue samples were conducted to determine *T. cruzi* infection status of adults as described in Ghersi et al. [8]. A total of 158 rodents were identified as *T. cruzi-*positive [8], including 12 parous females from three species (*Mus musculus, Rattus norvegicus,* and *Rattus rattus*) (Table 1). The 12 parous females were carrying a total of 66 embryos (Table 1). The number of embryos per mother ranged from two to 11, with parous females averaging approximately 5 embryos across all three species (Table 1). All embryos in this study were estimated to have developed for 13–17 days, where gestation period for rats and mice is approximately 21–23 days [29,30].

**Table 1:** Prevalence of *T. cruzi* infection in rodent offspring according to species and across all species; ± represents standard error of the mean.

Species	# Mothers	# Embryos (avg/ mother)	# Infected embryos	Embryo Infection prevalence
*R. norvegicus*	2	12 (6)	1	8.3% ± 8.3%
*R. rattus*	2	18 (9)	5	27.8% ± 10.5%
*M. musculus*	8	36 (4)	9	25% ± 7.2%
**All species**	12	66 (5.5)	15	22.7% ± 5.2%

### Tissue dissection and DNA extraction

Genomic DNA was extracted from all 66 embryos recovered from the *T. cruzi-*positive parous females. Up to 25 mg of embryonic tissue was used for genomic DNA extractions. Either the entire embryo was used for the extraction process or tissue was sampled from the thoracic region because the heart had not yet developed in any of the specimens used in this study. All dissections were carried out on a sterilized dissection pan in a Thermo Scientific Type A2 Biological Safety Cabinet (ThermoFisher Scientific, Waltham, MA). Embryos were dissected individually with sterilized forceps and scissors, first by removing the embryonic horn from the mother before separating each embryo from one another and placing each embryo on a different sterilized dissection pan. All tools and surfaces were sterilized with ethanol between each dissection. When the embryonic sac was intact, embryos were carefully removed by bisecting sac tissue to prevent cross-contamination between the embryo and mother. Forceps were used to peel embryonic sacks back away from embryos while a separate pair of forceps was used to extract the embryo from the bisected sac. All embryonic sacs and remaining embryonic tissues were placed into separate microcentrifuge tubes for long term storage at -80^o^ C. Genomic DNA was also obtained from 9 embryos carried by 2 *T. cruzi*-negative rodents to serve as negative controls. All DNA extractions were performed using Qiagen DNeasy Blood & Tissue Kits (QIAGEN, Valencia, CA) according to manufacturer protocols.

### Diagnostic and genotyping PCR assays

DNA extractions from all embryos were subjected to a sequence of PCR assays to first determine *T. cruzi* infection status and to then determine which of several possible DTUs were present in *T. cruzi*-positive individuals. The first diagnostic PCR assay, which relies on markers corresponding to highly repetitive genomic satellite DNA (described in [25]), allowed us to determine whether vertical transmission had occurred, and if so, its frequency within and among species. The subsequent genotyping PCR assay, which utilizes a primer multiplex based on the mini-exon gene to detect several DTUs (TCI, TCII, TCV) [31,32], allowed us to make a proof-of-principle determination of mixed infection. The DTUs detected by the genotyping multiplex are not exhaustive of all DTUs present in rodents, but nonetheless can provide evidence of mixed infection in offspring of *T. cruzi-*positive parous females. All PCR products were visualized using agarose gel electrophoresis with 2% gels cast with ethidium bromide.

### DNA sequence analysis

We further characterized the nature of *T. cruzi* infections by sequencing DTUs in *T. cruzi*-positive mothers and embryos. PCRs were conducted using primers TrypME and TcCH to amplify a 500 bp fragment of the mini-exon gene marker [33]. A total of 25 PCR products were generated from 10 mothers and 15 embryos. All products were purified using the Invitrogen PureLink PCR Purification Kit (Life Technologies, Carlsbad, CA). Following end-repair and indexing, libraries were prepared and sequenced on a MiSeq (Illumina) platform. Mini-exon sequences were mapped to reference sequences from each DTU to identify haplotypes using the FreeBayes [34] variant caller; sequences were deposited into GenBank under accession numbers PP933966-PP933986. Low abundance sequence variants (<0.1% of reads) were not included in the analysis. The reference sequences used were: Tcl: Raccoon70 (EF576837), Tcll:Tul8 (AY367125), TcIII: M5631(AY367126), TcIV: 92122102r (AY367124), TcV: SC43 (AY367127), TcVI: CL (U57984) and TcBat: TCC2477cll (KT305884). Additional sequence variants were identified using Geneious v9.1 (Dotmatics, Boston, MA). Maximum Likelihood phylogenetic trees were then built using MEGA X software [35]. Mini-exon sequences from other rodents from New Orleans [12] were included for comparison.

## Results

### Prevalence of vertical transmission

Overall, 15 of the 66 (22.7% ± 5.2%) embryos were PCR-positive for *T. cruzi* (Table 1). Evidence of vertical transmission was found for all three host species, with *T. cruzi* detected in embryos from 10 of the 12 *T. cruzi*-positive parous females included in the study (Fig 1). The frequency of vertical transmission ranged from 0% to 100%, averaging 32.9% per parous female. The frequency of vertical transmission also appears to differ by host species, with it being more frequent in house mice and black rats than Norway rats (Table 1).

### T. cruzi DTUs detected in embryos

PCR assays demonstrated that *T. cruzi*-positive embryos carried variable complements of DTUs and provided evidence of mixed infection. Of the 15 *T. cruzi*-positive embryos, one Norway rat embryo and five roof rat embryos displayed evidence of mixed infection of multiple DTUs associated with the TcI and non-TcI DTUs according to the genotyping PCR assay. Of the six embryos exhibiting evidence of mixed infection, all carried TcI as well as either TcII or TcV. The genotyping assay of mixed infection in house mice embryos was inconclusive.

We limited analyses of parasite sequence variation to two house mice embryos (Table 2) as sequences derived from all other samples could not be mapped to reference sequences. Sequence analysis recovered evidence of mixed infection in the two house mice embryos (Table 2). The recovered haplotypes from the two embryos corresponded to TcI (4 haplotypes) and TcV (3 haplotypes) in one of the embryos (Embryo 1609.6). The other embryo (Embryo 1793.2) carried TcII (one haplotype) and TcV (3 haplotypes).

**Table 2:** DTUs detected in *M. musculus* embryos and parous females captured in the study area as determined by deep sequencing.

Sample ID	Life stage	DTUs Present (haplotypes recovered)
**1609.6**	Embryo	TcI (4), TcII (1), TcVI (1), TcV (1)
**1793.2**	Embryo	TcI (1), TcII (1), TcV (2)
**1777**	Adult	TcV, TcVI (2)
**1779**	Adult	TcV (5)
**1773**	Adult	TcI

### DTU assemblage structure

**Fig 2:**
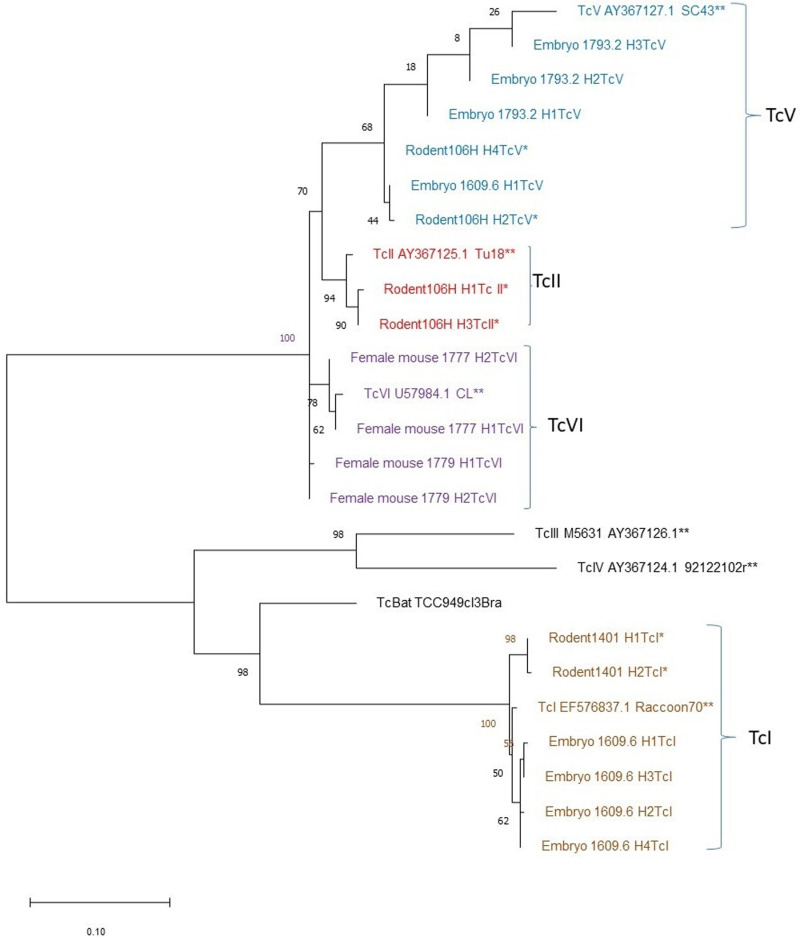
Evolutionary relationships among 25 specimens inferred according to the Maximum Likelihood method with a General Time Reversible model. The tree with the highest log likelihood (-3530.44) is shown. The tree is drawn to scale, with branch lengths measured in the number of substitutions per site. There was a total of 723 positions in the final dataset; numbers at nodes indicate bootstrap support. * indicate rodents captured from the same study area; ** indicate DTU reference sequences.

Direct comparisons of embryos to corresponding mothers were not possible, as sequences from the mothers of embryos 1609.6 and 1793.2 could not be mapped to reference sequences (Table 2). To gain further perspective on parasite diversity, we instead assessed the prevalence and haplotype diversity of DTU in both embryos and other parous female house mice with reference to data from other specimens captured in the study area. Notably, we recovered multiple haplotypes of TcV (mother 1779) and TcVI (mother 1777) in parous female house mice, which is consistent with previous work on *T. cruzi* variability in rodents from urban areas in Louisiana [12]. Maximum likelihood phylogenetic analysis (Fig 2) of parasite sequences recovered from infected parous females and embryos with reference to sequences from rodents and vectors from the study area- along with reference sequences from each DTU- indicates a broad parasite diversity, consistent across southern Louisiana.

## Discussion

### Evidence of vertical transmission

Mother-to-embryo comparisons provides the first indication that vertical transmission can occur in natural populations of rodent hosts inhabiting an urban landscape. We found evidence of potential vertical transmission across multiple platforms of molecular diagnostics in three common and widespread rodent host species at an overall transmission rate of 22.7%. The magnitude of mother-embryo transmission in Norway rats and roof rats was commensurate with rates observed in laboratory settings [22], while transmission in house mice was higher than had previously been detected under laboratory conditions [21]. The potential rates of transmission observed in our results would be higher than rates exhibited by other hosts such as rhesus macaques (3.9%) [36], Virginia opossum (2.7%) [37], and humans (4.7%) [38]. The rates of potential *T. cruzi* transmission in rodents we found would also be commensurate with other rodent-borne pathogens like *Toxoplasmosis gondii* (~18%) [39] and *Leishmania infantum* (~29%) [40]. Evidence of vertical transmission in natural populations of *T. cruzi* reservoirs indicates that our understanding of parasite-vector-host interactions warrants reconsideration. The persistence of *T. cruzi* infection among rodents via vertical transmission would be of particular concern considering that rodents are a widely distributed, prolific host of T. cruzi that come into frequent contact with humans [14,41–43].

Vector-borne transmission of *T. cruzi* has long been viewed as the primary- and arguably only- pathway of conveyance among non-human hosts [1]. Thus far, the importance of vertical transmission in non-humans has been discounted, in part because of clinical investigations [36] and work on reservoir species such as golden lion tamarin that did not detect evidence of vertical transmission [44]. Our study provides additional evidence supporting findings of laboratory-based studies on rodents. Moreover, our results indicate that vertical transmission could be a frequent occurrence in natural populations of common and widespread hosts like commensal rodents should offspring survive post-natal infection. If vertical transmission is indeed more common than previously believed, urban reservoirs may become larger and more permanent over time, posing greater risks to human health and well-being.

Our results suggest that vertical transmission could be an important mechanism for the maintenance of *T. cruzi* in hosts that inhabit landscapes that constrain interactions with vectors. In urban landscapes, for example, disassociation between *T. cruzi* vectors and hosts can occur because of habitat loss or chronic disturbance that limits the occurrence or depresses the prevalence of vectors. For instance, this may explain in part the limited success of a 3-year effort to detect triatomines in Houston (TX, USA) that only found nine individuals [17]. Habitat isolation and fragmentation can also limit movement and thus co-occurrence of hosts and vectors in urban landscapes. It follows then that vertical transmission might amplify the effects of limited interactions with *in situ* vectors or interactions in peripheral areas where vectors are more prevalent (e.g., peri-urban or neighboring sylvatic habitat). For example, evidence of vertical transmission has been found in stray dogs, which can be a widely distributed and common reservoir in cities [13,45]. It also follows that vertical transmission might supplant vector-borne transmission as the primary pathway in areas that provide for limited opportunities for direct interactions with vectors. Either scenario might allow for the maintenance and perhaps elevate the spread of infection, possibly increasing the risk of transmission to humans in areas that otherwise might not afford much opportunity for eco-epidemiological cycling.

### Evidence of mixed infection of embryos

We found evidence of mixed *T. cruzi* infections in embryos indicating that it is likely a common phenomenon in several species of commensal rodents. We had anticipated that mixed infection would be unlikely in embryos, particularly those that have not yet developed highly differentiated tissues, due to tissue tropism [24]. Rather, our study provides a benchmark finding of mixed infection in embryos from mothers that can carry diverse complements of *T. cruzi* DTUs. These results suggest that *T. cruzi* does not exhibit substantive tissue tropism and that DTUs are more likely paninfective [46]. The existence of embryos with mixed infections highlights the possibility that vertical transmission can contribute to the maintenance of *T. cruzi* genetic variation in natural reservoirs, especially considering the apparent rarity of sexual reproduction of *T. cruzi* in mammalian hosts [47,48]. Evidence of *T. cruzi* diversity being sustained across generations also points to the possibility of reservoir populations becoming self-sustaining where vectors are rare or absent (or after vectors have been removed). Moreover, diversity sustained over multiple generations could in part account for the heightened parasite diversity across the region (Fig 2), as has been explored in instances of vertical transmission in human mothers and children [49,50]. Maintenance of multiple DTUs via vertical transmission is also of concern because mixed infection can promote parasite persistence in hosts by decreasing virulence and potentially lead to more adverse human health outcomes over time [24,51].

### Conclusions

The impact of vertical transmission could be more muted than our results suggest in part because we did not account for offspring survival. The hemochorial placentation – wherein maternal blood can come into direct contact with developing offspring [36,52] – exhibited by rats and mice may account for the transmission of the parasite from mother to embryo during the developmental stage. Unlike prior investigations of vertical transmission in rodents, we examined embryos rather than pups born from *T. cruzi*-positive mothers. Some prior laboratory-based studies have tracked *T. cruzi* infection from the time of zygote formation to birth and through early pup development, while also noting secondary effects such as *T. cruzi* induced pup mortality [21]. Other approaches, such as testing wild female rodents accompanied by newborn offspring [37], can circumvent potential limiting factors such as contamination of embryos during dissection.

Accordingly, it would be prudent to mount further investigations to better understand the impact of vertical transmission on *T. cruzi* prevalence and diversity in natural reservoir populations. For example, combining diagnostic and genotyping assays with genomic estimates of relatedness among *T. cruzi*-positive individuals could shed further light on the importance of vertical transmission in natural populations. Further work is warranted to obtain definitive evidence of vertical transmission in natural populations of rodents, such as testing wild female rodents accompanied by newborn offspring, as has been done in recent work on Virginia opossum [37]. Broader ecological assessments of infection prevalence versus habitat isolation or fragmentation could likewise provide valuable perspectives on the relative importance of vertical transmission, particularly in urban areas where interactions with triatomine vectors are likely to be uncommon. Insights gained from mounting additional investigations could afford a stronger basis for mitigating the risk of transmission to humans where Chagas disease is widespread or emerging as a public health concern.
